# Effects of Phosphoethanolamine Supplementation on Mitochondrial Activity and Lipogenesis in a Caffeine Ingestion *Caenorhabditis elegans* Model

**DOI:** 10.3390/nu12113348

**Published:** 2020-10-30

**Authors:** Hyemin Min, Esther Youn, Jaehoon Kim, Su Young Son, Choong Hwan Lee, Yhong-Hee Shim

**Affiliations:** 1 Department of Bioscience and Biotechnology, Konkuk University, Seoul 05029, Korea; mintmin@konkuk.ac.kr (H.M.); dptmej@konkuk.ac.kr (E.Y.); kb2959@konkuk.ac.kr (J.K.); syson119@naver.com (S.Y.S.); chlee123@konkuk.ac.kr (C.H.L.); 2 Research Institute for Bioactive-Metabolome Network, Konkuk University, Seoul 05029, Korea

**Keywords:** caffeine, 1,3,7-trimethylxanthine, phosphoethanolamine, mitochondrial activity, oxidative stress response, AMP-activated protein kinase, lipogenesis, *Caenorhabditis elegans*

## Abstract

Caffeine intake is strongly linked to lipid metabolism. We previously reported the age-dependent physiological effects of caffeine intake in a *Caenorhabditis elegans* model. Since nutritional status can actively influence metabolism and overall health, in this study, we evaluated the effect of caffeine intake on lipid metabolism in adult-stage *C. elegans*. We found that, in *C. elegans*, fat storage and the level of phosphoethanolamine (PE) were significantly reduced with caffeine intake. In addition, mitochondrial activity decreased and mitochondrial morphology was disrupted, and the expression of oxidative stress response genes, *hsp-6*, *gst-4*, and *daf-16*, was induced by caffeine intake. Furthermore, the level of an energy metabolism sensor, phospho-AMP-activated protein kinase, was increased, whereas the expression of the sterol regulatory element binding protein gene and its target stearoyl-CoA desaturase genes, *fat-5*, *-6*, and *-7*, was decreased with caffeine intake. These findings suggest that caffeine intake causes mitochondrial dysfunction and reduces lipogenesis. Interestingly, these changes induced by caffeine intake were partially alleviated by PE supplementation, suggesting that the reduction in mitochondrial activity and lipogenesis is in part because of the low PE level, and proper dietary supplementation can improve organelle integrity.

## 1. Introduction

Caffeine is the most popular drug consumed worldwide; approximately 80% of the world’s population consumes caffeinated foods every day [1,2]. Caffeine is rapidly absorbed through the gastrointestinal tract, moves through cellular membranes [3], is metabolized in the liver, and results in three metabolites: paraxanthine, theophylline, and theobromine [1,3]. Lipid accumulation and metabolism are dependent on the presence and production of intracellular second messenger molecules such as cyclic adenosine monophosphate (cAMP). The increased cAMP response is short-lived because it is rapidly degraded by phosphodiesterase (PDE). The intracellular signal can be sustained for a longer period by the inhibition of PDEs such as methylxanthines that are present in caffeine. Caffeine has also been associated with loss in body weight and increased energy expenditure in humans and animal models [4,5,6,7]. These studies indicate the strong relationship between caffeine intake and lipid metabolism. However, it is largely unknown how caffeine intake modulates various physiological processes in animals including lipid metabolism at the molecular level.

*Caenorhabditis elegans* is an excellent animal model to study the metabolic effects of nutrient intake on the body because it is easy to maintain in large populations and convenient for organ observation owing to its transparent body. Furthermore, *C. elegans* offers a relevant model to elucidate the molecular mechanisms of metabolic regulation that are conserved in mammals, which would lead to the understanding of the basis of metabolic disorders [8,9]. Although several studies have reported that caffeine intake has both beneficial and adverse effects on *C. elegans* development, reproduction, and aging in an age- and dose-dependent manner [10,11,12,13,14,15,16], the effects on its metabolism are still elusive.

In this study, we investigated the control of lipid metabolism by 10 mM of caffeine intake in adult-stage *C. elegans* and its association with mitochondrial activity. We observed reductions in fat storage, phosphoethanolamine (PE) level, and mitochondrial activity. Expression of oxidative stress response genes, *hsp-6* and *gst-4* and the level of phospho-AMPK were increased, and DAF-16, which is activated by phospho-AMPK to respond to metabolic stress, was activated by nuclear localization. It appears that the phospho-AMPK activated by caffeine intake also decreases lipogenesis by reducing the expression of *sbp-1*, an ortholog of *C. elegans* sterol regulatory element binding protein (SREBP) gene and its target stearoyl-CoA desaturase (SCD) genes, *fat-5*, *fat-6*, and *fat-7*. We further investigated the effects of PE supplementation with caffeine intake on mitochondrial activity and stress responses and found that the changes caused by caffeine intake were alleviated by PE supplementation. Taken together, this study suggests that caffeine intake reduces the level of PE, induces mitochondrial stress, and causes an energy imbalance, which induces the AMPK-mediated stress response and reduces fat storage. In addition, our findings suggest that the PE level is a key component in maintaining mitochondrial integrity.

## 2. Materials and Methods

### 2.1. Caenorhabditis elegans Strains and Treatment with Caffeine

*C. elegans* strains were maintained at either 15 or 20 °C on nematode growth medium (NGM) agar plates seeded with *Escherichia coli* strain OP50, as described previously [17]. The following strains were used in the present study: N2 (*C. elegans* wild isolate, Bristol variety), SJ4103: *zcIs14 [myo-3::GFP(mit)]*, RW1596: *myo-3(st386) V*; *stEx30 [myo-3p::GFP::myo-3+rol-6(su1006)]*, SJ4005: *zcIs4 [hsp-4::GFP]*, SJ4100: *zcIs13 [hsp-6::GFP]*, TJ375: *gpIs1 [hsp-16.2p::GFP]*, CL2166: *dvIs19 [(pAF15)gst-4p::GFP::NLS]III*, GR1352: *xrIs87 [daf-16(alpha)::GFP::daf-16B+rol-6(su1006)]*, CE548: *sbp-1(ep79) III*; *epEx141 [sbp-1::GFP::SBP-1+rol-6(su1006)]*, BX150: *lin-5B&lin-15A(n765) X*; *waEx18 [fat-5::GFP+lin15(+)]*, BX115: *lin-5B&lin-15A(n765) X*; *waEx16 [fat-6::GFP+lin15(+)]*, and BX113: *lin-5B&lin-15A(n765) X*; *waEx15 [fat-7::GFP+lin15(+)]*. To examine the effects of caffeine intake, 10 mM of caffeine (Sigma-Aldrich, St. Louis, MO, USA) was added to NGM before autoclaving, as previously described [13]. Synchronized L4-stage animals were exposed to caffeine for 24 h at 20 °C, and then the adult-stage animals were examined.

### 2.2. Analysis of Lipid Composition Using Gas Chromatography-Time of Flight-Mass Spectrometry (GC-TOF-MS)

Sample preparation for the analysis of lipid composition in *C. elegans* was performed as previously described with minor modifications [18]. Briefly, L4-stage wild-type N2 hermaphrodites were individually cloned onto either caffeine-containing (10 mM) or caffeine-free (0 mM) NGM agar plates and grown for 24 h at 20 °C. Five thousand adult animals from each treatment were collected in M9 buffer and washed three times. After centrifugation, the M9 buffer was removed, and the samples were frozen in liquid nitrogen. Each sample was extracted with 80% methanol and internal standard solution (2-chloro-phenylalanine, 1 mg/mL in water) using an MM400 mixer mill (Retsch^®^, Haan, Germany) at a frequency of 30 s^−1^ for 10 min, followed by sonication for 10 min. Subsequently, the extracted samples were centrifuged, and the supernatants were filtered through 0.2 μm polytetrafluoroethylene filters (Chromdisc, Daegu, Korea). The filtered samples were completely evaporated using a speed vacuum concentrator (Biotron, Seoul, Korea). The final concentration of each sample was 10 mg/mL in methanol for MS analysis.

GC-TOF-MS analysis was performed as previously reported [19]. The dried samples were oximated with methoxyamine hydrochloride (20 mg/mL in pyridine) for 90 min at 30 °C and silylated with MSTFA for 30 min at 37 °C. Finally, the samples were injected into a GC-TOF-MS instrument (Thermo Fisher Scientific, West Palm Beach, FL, USA) in splitless mode. MS data analysis and multivariate statistical analysis were conducted as previously described [19]. Selected metabolites derived from GC-TOF-MS data were tentatively identified using standard compounds and comparisons of their retention time and MS fragments. We also confirmed the MS spectrum data for selected metabolites with available databases including the National Institute of Standards and Technology (NIST) database (Version 2.0, 2011, FairCom, Gaithersburg, MD, USA), Wiley 9, and the Human Metabolome Database (HMDB; http://www.hmdb.ca). Significant differences between groups (*p*-value) were evaluated using PASW Statistics (IBM SPSS Inc., Chicago, IL, USA).

### 2.3. Analysis of Mitochondrial Activity

L4-stage wild-type N2 hermaphrodites were treated with caffeine for 24 h at 20 °C. The adult-stage animals were then incubated for 4 h at 20 °C with 10 μM of MitoTracker Red (Invitrogen, Carlsbad, CA, USA), which is a fluorescent probe that accumulates in active mitochondria. After MitoTracker staining, the animals were immobilized using 0.2 mM tetramisole hydrochloride (Sigma-Aldrich, St. Louis, MO, USA) in M9 buffer and mounted on a poly-l-lysine- (Sigma-Aldrich, St. Louis, MO, USA) coated glass slide. Live images of stained animals were observed under a fluorescence microscope (Zeiss Axioscope, Oberkochen, Germany), and the average pixel intensity of MitoTracker Red fluorescence was measured using ImageJ.

### 2.4. Live Image Observation of Fluorescence-Tagged Transgenic Animals

The synchronized L4-stage transgenic animals expressing GFP were treated with caffeine for 24 h at 20 °C. The animals were then immobilized using 0.2 mM tetramisole hydrochloride (Sigma-Aldrich, St. Louis, MO, USA) in M9 buffer and mounted on a poly-l-lysine- (Sigma-Aldrich, St. Louis, MO, USA) coated glass slide. Live images of animals were observed under a fluorescence microscope (Zeiss Axioscope, Oberkochen, Germany). Using ImageJ, we measured the average pixel intensity of fluorescence in GFP-tagged transgenic animals including SJ4103, RW1596, SJ4005, SJ4100, TJ375, CL2166, GR1352, CE548, BX150, BX115, and BX113 for quantification.

### 2.5. Analysis of Mitochondrial Reactive Oxygen Species (ROS) and Superoxide Levels Using Fluorescence Microscopy

To examine the effect of caffeine intake on mitochondrial ROS and superoxide levels, CellROX^®^ Green (Invitrogen, Carlsbad, CA, USA) and MitoSOX (Invitrogen, Carlsbad, CA, USA) staining were performed as previously described [20,21]. CellROX^®^ Green and MitoSOX were freshly prepared at 5 mM for the stock solution and diluted in M9 buffer at a 1:1000 dilution before treatment. Synchronized adult-stage animals fed with either a caffeine-free or a caffeine diet were transferred into the staining solution and stained for 20 min at 20 °C. Animals were mounted on a poly-l-lysine- (Sigma-Aldrich, St. Louis, MO, USA) coated glass slide and then observed under a fluorescence microscope (Zeiss Axioscope, Oberkochen, Germany). Relative quantitation of mitochondrial ROS and superoxide levels was measured using ImageJ.

### 2.6. Western Blot Analysis

Western blot analysis was performed as previously described [22]. Whole animal protein extract prepared from 300 gravid adult hermaphrodites from each treatment was used per gel well. Antibodies bound to a nitrocellulose membrane (PROTRAN BA83, Whatman, Sigma-Aldrich, St. Louis, MO, USA) were visualized using an ECL Western blotting detection kit (Amersham, GE Healthcare Life Sciences, Pittsburgh, PA, USA), and the respective band intensities were measured using a LAS-3000 image analyzer with Multi Gauge (v.3.0) (Fuji Film, Tokyo, Japan). The following primary and secondary antibodies were used: rabbit anti-AMPK (1:1000, Cell Signaling Technology, Danvers, MA, USA), rabbit anti-pAMPK (1:1000, Cell Signaling Technology, Danvers, MA, USA), mouse anti-α-tubulin (1:1000; Sigma-Aldrich, St. Louis, MO, USA), HRP-conjugated goat anti-rabbit IgG (1:1000; Santa Cruz Biotechnology, Dallas, TX, USA), and HRP-conjugated donkey anti-mouse IgG (1:1000; Jackson ImmunoResearch, West Grove, PA, USA).

### 2.7. Analysis of Body Fat Using Nile Red (NR) and Oil Red O Staining

Body fat content was measured as previously described [23]. In brief, a stock solution was prepared by dissolving NR (Invitrogen, Carlsbad, CA, USA) in acetone (5 mg/mL). Synchronized adult-stage animals were treated with 40% isopropanol for 3 min at 20 °C, and NR working solution (30 μg/mL) was added to each sample for 2 h at 20 °C in the dark. For Oil Red O staining, a stock solution was prepared by dissolving Oil Red O (500 mg) in 100% isopropanol (100 mL), and the stock solution was diluted in water (3:2) to 60% isopropanol, and filtered with a 0.2 μm sterile syringe filter. Synchronized adult-stage animals were treated with 40% isopropanol for 3 min at 20 °C, and Oil Red O solution was added to each sample for 2 h at 20 °C in the dark. Body fat content was measured in lipid droplets of NR-stained or Oil Red O-stained animals using a Zeiss microscope at 20× magnification. Using ImageJ, we measured the average pixel intensity for the quantification of NR and Oil Red O staining. For each case, three independent experiments were performed.

### 2.8. Supplementation with PE and Ethanolamine (ETA)

PE and ETA were purchased from Sigma-Aldrich (St. Louis, MO, USA). PE or ETA was added to autoclaved NGM before pouring into agar plates. The synchronized L4-stage animals were placed onto NGM agar media supplemented with either PE (0.1 mM to 50 mM) or ETA (5 mM) and with or without 10 mM of caffeine for 24 h at 20 °C and subjected to the respective experiments.

### 2.9. Statistical Analysis

All experiments were repeated more than three times for statistical evaluation of the data. The *p* values were calculated using either two-tailed Student’s *t*-test or one-way ANOVA test. *p* < 0.05 was considered significant. The data are expressed as the mean ± standard deviation (SD).

## 3. Results

### 3.1. Reduction in the Level of PE and Mitochondrial Activity, and the Disruption of Mitochondrial Morphology with Caffeine Intake in C. elegans

**Figure 1:**
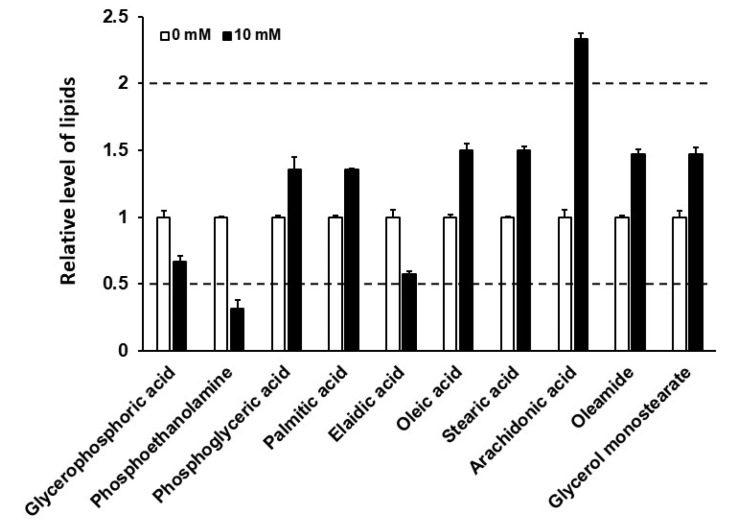
Caffeine intake altered the level of lipid composition in *Caenorhabditis elegans*. Fold change in fatty acids between the caffeine-free diet group (control, white bar) and the caffeine-fed group (black bar), as determined by gas chromatography. Fatty acids were extracted from 5000 adult animals for each group. The dotted lines indicate a relative difference of more than 2-fold compared to the control. Significant differences between groups (*p*-value) are shown in Supplementary Table S1. Error bars represent standard deviation (SD).

Previous studies have suggested that caffeine intake affects lipid metabolism [7,24,25]. Therefore, we attempted to evaluate lipid metabolism regulation by caffeine intake in *C. elegans*. GC-TOF-MS chromatography was performed to analyze changes in fatty acid composition owing to caffeine intake. The levels of glycerophosphoric acid, phosphoglyceric acid, palmitic acid, elaidic acid, oleic acid, stearic acid, oleamide, and glycerol monostearate were significantly altered with less than 2-fold differences (*p* < 0.05) compared to the levels in the caffeine-free diet control group; however, the levels of PE and arachidonic acid (AA) showed more than a 2-fold decrease or increase, respectively (Figure 1 and Supplementary Table S1, *p* < 0.05). The most drastic reduction in lipid compositions by caffeine intake was PE based on the analysis by GC-TOF-MS chromatography (Figure 1). This result suggests that the low level of PE causes major effects of caffeine intake in *C. elegans*. Therefore, further investigation was focused on the effects of PE after caffeine intake. PE is a major component of the mitochondrial inner membrane where the energy production process occurs [26,27].

**Figure 2:**
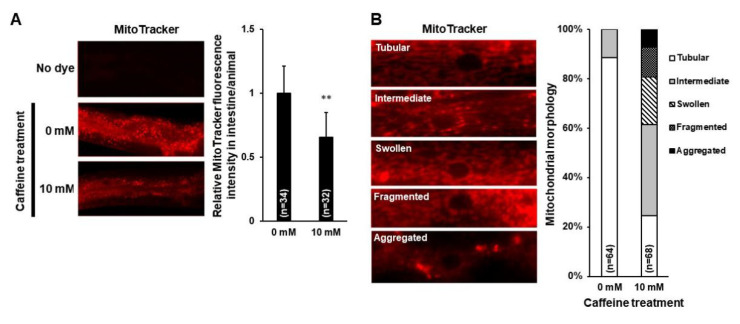

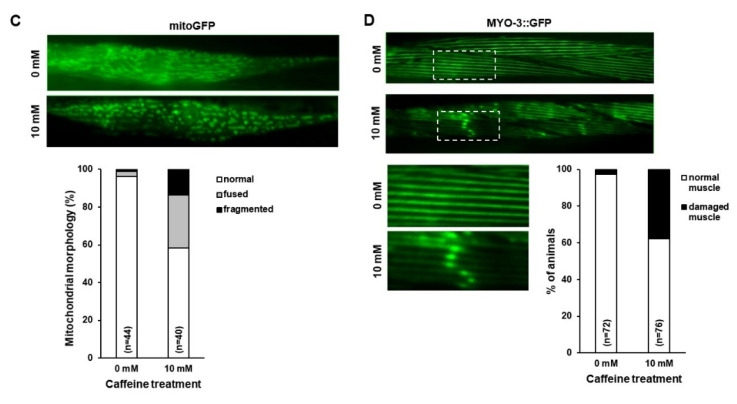
Caffeine intake reduced mitochondrial activity and disrupted mitochondrial morphology in *Caenorhabditis elegans*. (**A**) Comparison of mitochondrial activity in the intestine of caffeine-fed animals (10 mM) and that of the caffeine-free diet control group (0 mM) using MitoTracker staining. The graph shows the relative level of mitochondrial activity using ImageJ analysis. Statistical significance was calculated using Student’s *t*-test. **, *p* < 0.01. Error bars represent standard deviation. (**B**) Comparison of mitochondrial morphology in the muscle cells of caffeine-fed animals (10 mM) and that of the caffeine-free diet control group (0 mM) using MitoTracker staining. The graph indicates the percentage of animals with mitochondria classified as tubular, intermediate, fragmented, swollen, or aggregated. (**C**) The effect of caffeine intake on mitochondrial morphology was analyzed in the transgenic animal SJ4103 expressing a mitochondrial-targeted GFP under control of the muscle-specific *myo-3* promoter. The graph indicates the percentage of animals with muscle mitochondria classified as indicated. (**D**) Myofilament abnormality owing to caffeine intake was visualized using the MYO-3::GFP transgene. Representative fluorescent images in the caffeine-free diet control group (0 mM) showed normal filament organization. MYO-3::GFP abnormalities (aggregations) were observed in the caffeine-fed animals (10 mM). The graph indicates the percentage of animals classified as having normal muscle or damaged muscle (aggregations).

To investigate the possible alterations in mitochondria owing to the low level of PE in caffeine-fed animals, we examined both mitochondrial activity in the intestine and morphological changes in the muscle cells of *C. elegans* using MitoTracker staining (Figure 2A,B). In the caffeine-free diet animals, the mitochondrial activity in the intestine was normal, and the majority of muscle mitochondria showed a tubular morphology (Figure 2A,B). In contrast, the animals that ingested caffeine showed a significant decrease in mitochondrial activity in the intestine (*p* < 0.05) and alterations in mitochondrial morphology, including fragmentation (12.2% ± 1.2), swelling (19.4% ± 2.1), and aggregation (6.9% ± 0.8) in the muscle cells (Figure 2A,B). We confirmed the alterations in mitochondrial morphology owing to caffeine intake using transgenic animals containing the transgene, *Pmyo-3::mitoGFP*, which is expressed in the mitochondria of the muscle cells (Figure 2C). Furthermore, the morphological changes in mitochondria leading to damage in muscle cells of caffeine-fed animals were also observed using a transgene, MYO-3::GFP, in the muscle cells (Figure 2D). These results indicated that caffeine intake decreased the mitochondrial activity and disrupted mitochondrial morphology.

### 3.2. Caffeine Intake Activates AMPK and DAF-16 by Inducing Mitochondrial Stress Response in C. elegans

**Figure 3:**
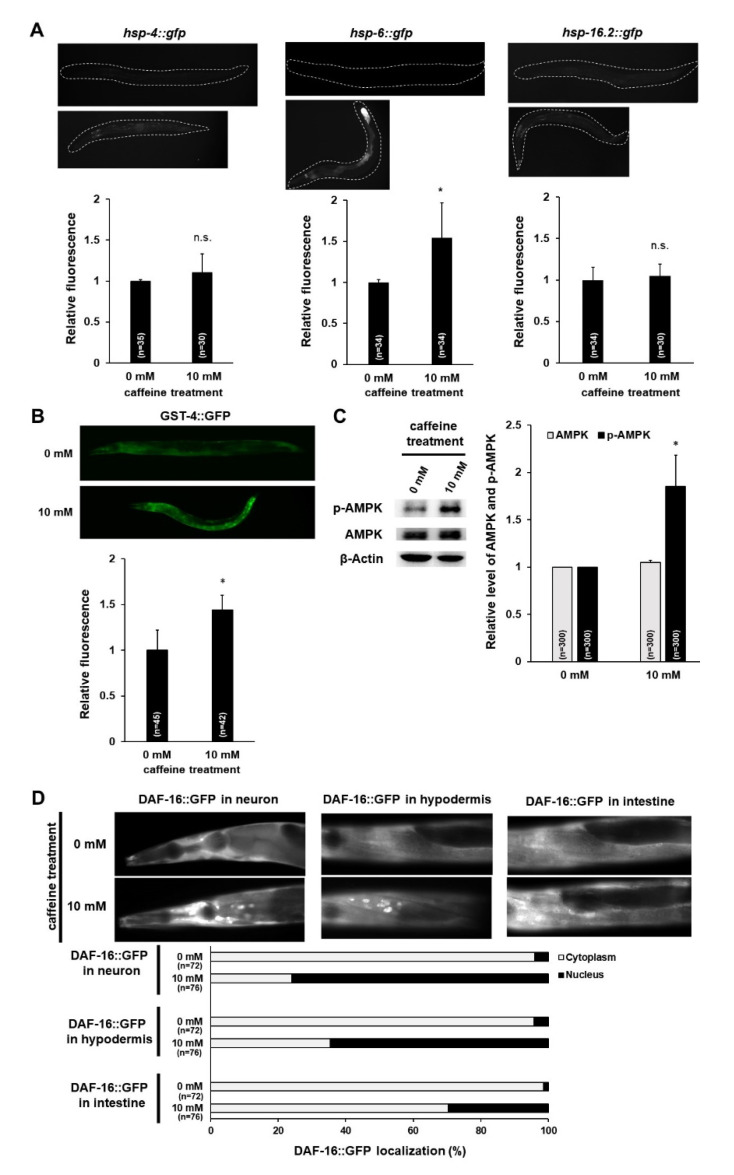
Caffeine intake activates AMPK and DAF-16 by inducing the mitochondrial stress response in *Caenorhabditis elegans*. (**A**,**B**) HSP-4::GFP, HSP-6::GFP, HSP-16.2::GFP, and GST-4::GFP transgenic animals synchronized at the L4-stage were fed either 0 or 10 mM of caffeine for 24 h at 20 °C, and respective fusion proteins were observed under fluorescence microscopy and quantified using ImageJ analysis. Statistical significance was calculated using Student’s *t*-test. *, *p* < 0.05. n.s., *p* > 0.05. Error bars represent standard deviation (SD). (**C**) Western blot analysis of phospho-AMPK protein levels in animals fed the caffeine-free diet (0 mM) or caffeine diet (10 mM). AMPK and phospho-AMPK band intensities were normalized against those of β-Actin in the same lane. Then, the normalized AMPK and p-AMPK band intensities were converted to a relative value compared to the normalized AMPK and p-AMPK band intensities of 0 mM, as shown in the graph; values are mean ± SD of three biological replicates of lysates from 300 animals. Statistical significance was calculated using Student’s *t*-test. * *p* < 0.05. (**D**) Images show DAF-16::GFP expression pattern in both caffeine-free diet animals and caffeine-fed animals in the respective tissues. The graph indicates the percentage of animals that show the distribution of subcellular localization of DAF-16 in the respective tissues.

It was previously reported that changes in mitochondrial activity and morphology are associated with cellular stress responses as a cellular adaptation to regulate cell survival [28]. In addition, mitochondrial damage can induce unfolded protein stress responses [29,30] and mitochondrial morphology can play a role in mitochondrial energetics in *C. elegans* [31,32]. To examine whether the mitochondrial changes owing to caffeine intake affect cellular stress responses, we investigated transgenic animals expressing a gene involved in three different stress response pathways, including an endoplasmic reticulum chaperone (*hsp-4*), a mitochondrial chaperone (*hsp-6*), and a cytosolic chaperone (*hsp-16.2*), and quantified the relative protein levels of each reporter (Figure 3A). Among three cellular stress responses, caffeine-fed adult-stage transgenic animals expressing HSP-6 showed significantly increased expression levels of GFP fluorescence compared to those in the caffeine-free diet control group, suggesting that caffeine intake induces mitochondrial stress response (Figure 3A). Furthermore, we found that caffeine intake increased the level of *gst-4* expression, an antioxidant enzyme, in transgenic animals expressing the *Pgst-4::GFP* gene (Figure 3B). This finding suggests that caffeine intake induces the oxidative stress response. In addition, we determined the levels of ROS and superoxide in mitochondria using CellROX Green and MitoSox Red, the fluorescence dyes that have been used to detect the mitochondrial ROS and mitochondrial superoxide, respectively. MitoSox Red, a mitochondrial superoxide indicator failed to stain in caffeine-fed animals (Supplementary Figure S1). However, CellROX staining showed the increased level of mitochondrial ROS in caffeine-fed animals while only the basal level of mitochondrial ROS was detected in caffeine-free diet animals (Supplementary Figure S2). These results indicate that caffeine intake activates mitochondrial oxidative stress response by elevating mitochondrial ROS but not by superoxide.

The correlation between mitochondrial functions and AMPK under dietary stress conditions in *C. elegans* has been reported previously [33,34,35]. Therefore, by performing Western blot, we next examined whether caffeine intake affects the activation of AMPK. We found that caffeine intake indeed activated AMPK by increasing the level of phosphorylated AMPK (p-AMPK) (Figure 3C). We further assessed a possible association by caffeine intake between the activation of AMPK and DAF-16, a direct target of phospho-AMPK, as a key transcription factor in the insulin/IGF signaling pathway [36,37]. The *daf-16* gene encodes a FOXO transcription factor, which is expressed in the cytoplasm and localized to the nuclei when activated in most cells including the neurons, muscles, hypodermis, and intestine in *C. elegans* [38,39,40]. We compared the subcellular localization of DAF-16::GFP transgene in both caffeine-free diet controls and caffeine-fed animals (Figure 3D). Caffeine-fed transgenic animals showed significant DAF-16::GFP nuclear localization in the neurons, hypodermis, and intestine (Figure 3D). Taken together, we suggest that caffeine intake induces the mitochondrial stress response and increases *gst-4* expression, which activates AMPK phosphorylation and promotes DAF-16 nuclear localization.

### 3.3. Caffeine Intake Reduces Lipogenesis and Fat Storage in C. elegans

**Figure 4:**
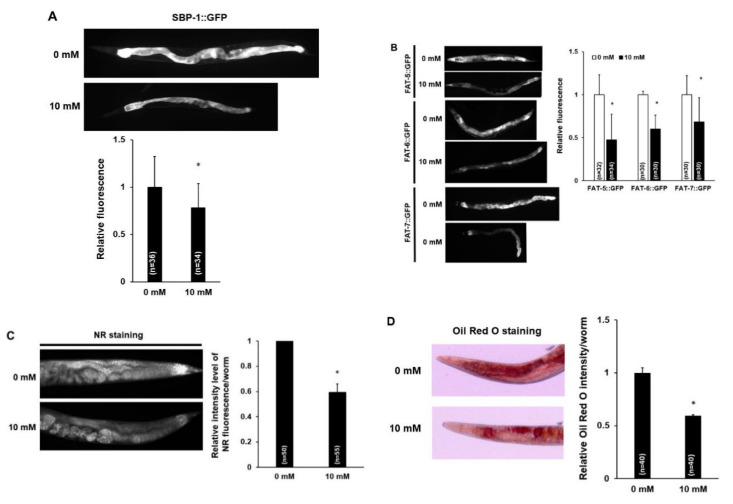
Caffeine intake suppresses lipogenesis and fat storage in *Caenorhabditis elegans*. (**A**) SBP-1::GFP transgenic animals synchronized at the L4-stage were exposed to caffeine (10 mM) for 24 h at 20 °C. A reduced level of SBP-1::GFP in the intestine was observed in caffeine-fed animals. Statistical significance was calculated using Student’s *t*-test. *, *p* < 0.05. Error bars represent standard deviation (SD). (**B**) FAT-5::GFP, FAT-6::GFP, and FAT-7::GFP transgenic animals synchronized at the L4-stage were exposed to caffeine (10 mM) for 24 h at 20 °C. The caffeine-fed transgenic animals showed reduced intensity levels of FAT-5::GFP, FAT-6::GFP, and FAT-7::GFP in the intestine. The graph indicates the effect of caffeine intake on the relative levels of fusion protein intensity values in the respective transgenic animals. Statistical significance was calculated using Student’s *t*-test. *, *p* < 0.05. Error bars represent SD. (**C**,**D**) Caffeine-fed animals displayed reduced lipid content as quantified by Nile Red (NR) and Oil Red O staining. Wild-type animals synchronized at the L4-stage were exposed to caffeine (10 mM) for 24 h at 20 °C. The graphs show the relative NR fluorescence or Oil Red O intensity in caffeine-fed animals compared to those in animals fed a caffeine-free diet. Statistical significance was calculated using Student’s *t*-test. *, *p* < 0.05. Error bars represent SD.

AMPK phosphorylation has been implicated in the regulation of multiple metabolic processes in cells, including sterol regulatory element binding protein (SREBP), a master transcriptional regulator of lipid synthesis [41,42]. SCD is a key target of SREBP [43,44,45,46], and there are three *C. elegans scd* orthologs, *fat-5*, *fat-6*, and *fat-7* [47,48]. Therefore, we examined the levels of *sbp-1*, a *C. elegans* ortholog of human SREBP, and its target genes using transgenic animals expressing *sbp-1*, *fat-5*, *fat-6*, and *fat-7* (Figure 4A,B). Caffeine-fed transgenic animals showed significantly decreased expression of not only SBP-1::GFP, but also FAT-5, -6, and -7::GFP (Figure 4A,B). These results indicated that caffeine intake reduced lipogenesis.

As caffeine-fed animals showed the down-regulated expression of lipogenesis genes, *sbp-1*, *fat-5*, *fat-6*, and *fat-7*, we further examined fat storage in these animals with both NR and Oil Red O staining, the dyes that selectively stain lipid droplets in the cells. The fat storage was decreased in wild type N2 animals fed with caffeine compared to that in animals fed the caffeine-free diet (Figure 4C,D), indicating that caffeine intake suppresses fat storage in *C. elegans*.

### 3.4. PE Supplementation Improves Mitochondrial Activity and Morphology in Caffeine-Fed Animals

**Figure 5:**
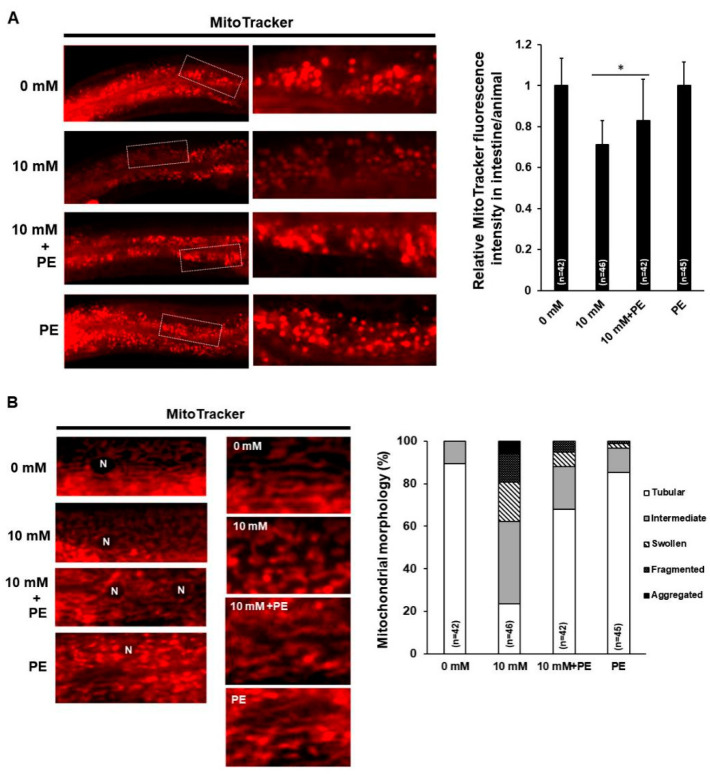

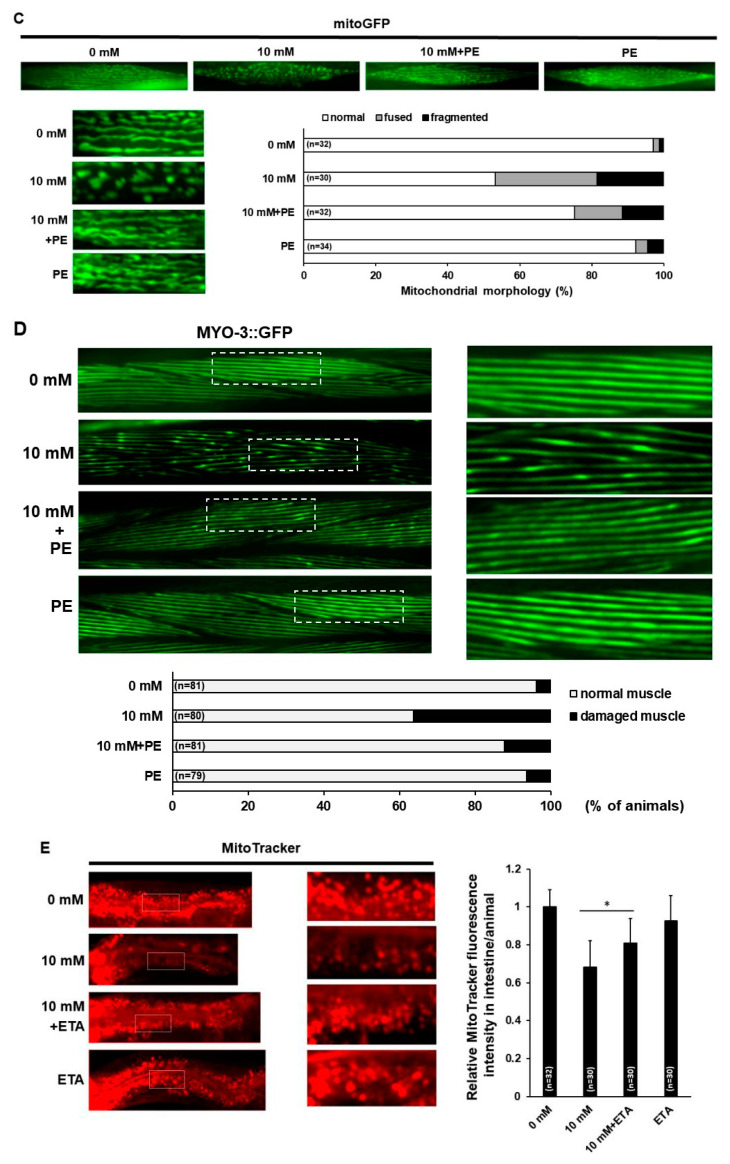

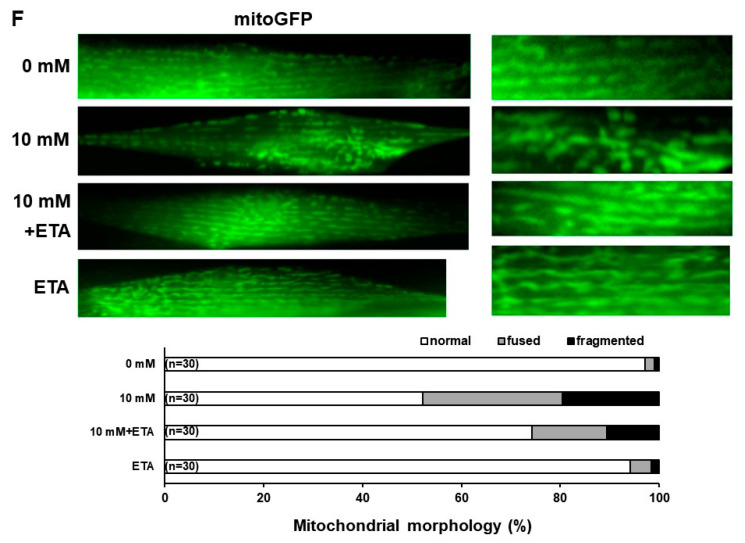
Supplementation with phosphoethanolamine (PE) or ethanolamine (ETA) mitigated the altered mitochondrial activity and morphology owing to caffeine intake in *Caenorhabditis elegans*. (**A**) Comparison of mitochondrial activity in the intestine of animals fed caffeine supplemented with PE with that of caffeine-fed animals using MitoTracker staining. The graph shows the relative levels of mitochondrial activity using ImageJ analysis. Statistical significance was calculated using one-way ANOVA. * *p* < 0.05. Error bars represent standard deviation (SD). (**B**) Comparison of mitochondrial morphology in the muscle cells of animals fed caffeine supplemented with PE with that of caffeine-fed animals using MitoTracker staining. The graph indicates the percentage of animals with mitochondria classified as tubular, intermediate, fragmented, swollen, or aggregated. (**C**) The effect of caffeine intake with PE supplementation on mitochondrial morphology was analyzed in the transgenic strain SJ4103 expressing a mitochondrial-targeted GFP under the control of the muscle-specific *myo-3* promoter. The graph indicates the percentage of animals with muscle mitochondria classified as normal, fused, or fragmented. (**D**) Myofilament abnormality owing to caffeine intake visualized using the MYO-3::GFP transgene. Representative fluorescent images are shown for both the caffeine-free diet condition (0 mM) and PE supplementation condition (PE), resulting in normal filament organization. The MYO-3::GFP abnormalities (aggregations) observed in caffeine-fed animals (10 mM) were restored by PE supplementation. The graph indicates the percentage of animals classified as having normal muscle or damaged muscle (aggregations) in the respective conditions. (**E**) Comparison of mitochondrial activity in the intestine of animals fed caffeine supplemented with ETA with that of caffeine-fed animals using MitoTracker staining. The graph shows the relative levels of mitochondrial activity using ImageJ analysis. Statistical significance was calculated using one-way ANOVA. *, *p* < 0.05. Error bars represent SD. (**F**) The effect of caffeine intake with ETA supplementation on mitochondrial morphology was analyzed in the transgenic strain SJ4103 expressing a mitochondrial-targeted GFP under the control of the muscle-specific *myo-3* promoter. The graph indicates the percentage of animals with muscle mitochondria classified as normal, fused, or fragmented.

Dietary intake of phospholipids is known to have beneficial effects on several chronic diseases, including heart disease, inflammation, cancer, metabolic disease, and health promotion [49]. We investigated the effects of PE supplementation to determine whether PE supplementation alleviates the mitochondrial dysfunction induced by caffeine intake. We examined the effects of PE supplementation at concentrations of 0.1 mM, 1 mM, 5 mM, 10 mM, or 50 mM on mitochondrial activity in the intestine of *C. elegans* (Supplementary Figure S2). We found that PE supplementation significantly improved the mitochondrial activity that was decreased by caffeine intake and its effect was saturated at 5 mM of PE (Supplementary Figure S2 and Figure 5A), suggesting that PE supplementation has a beneficial effect on mitochondrial activity under caffeine diet conditions and that supplementation with 5 mM PE is optimal.

To confirm whether the PE supplementation also improves the mitochondrial morphology disrupted by caffeine intake, we analyzed the mitochondrial morphology and the integrity of muscle cells with PE supplementation through both MitoTracker staining and transgenic animals expressing the transgene, *Pmyo-3::mitoGFP* or *myo-3::GFP* (Figure 5B–D). PE supplementation also significantly improved mitochondrial morphology in caffeine-fed animals (Figure 5B–D), indicating that PE is an important component for mitochondrial activity and morphology and PE supplementation can overcome mitochondrial defects induced by caffeine intake.

We investigated the effects of supplementation with ethanolamine (ETA), a precursor of PE, on mitochondrial activity and morphology in caffeine-fed animals. ETA supplementation also alleviated the decrease in mitochondrial activity in the intestine and the disruption in mitochondrial morphology in the muscle cells owing to caffeine intake (Figure 5E,F). Taken together, our findings clearly support the premise that the decrease in PE level induced by caffeine intake leads to defects in mitochondrial activity and morphology.

### 3.5. PE Supplementation Suppresses the Activation of AMPK and DAF-16 Induced by Caffeine Intake in C. elegans

**Figure 6:**
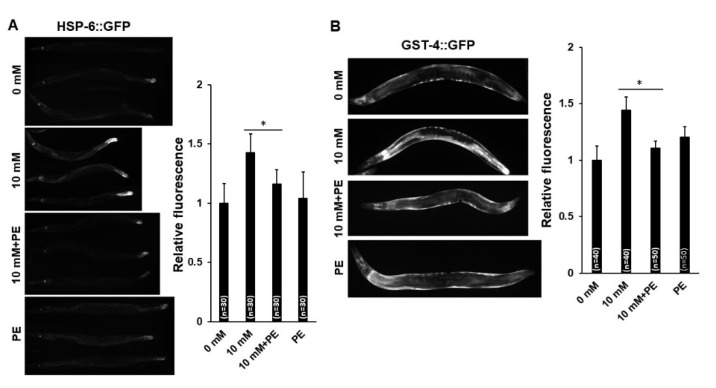

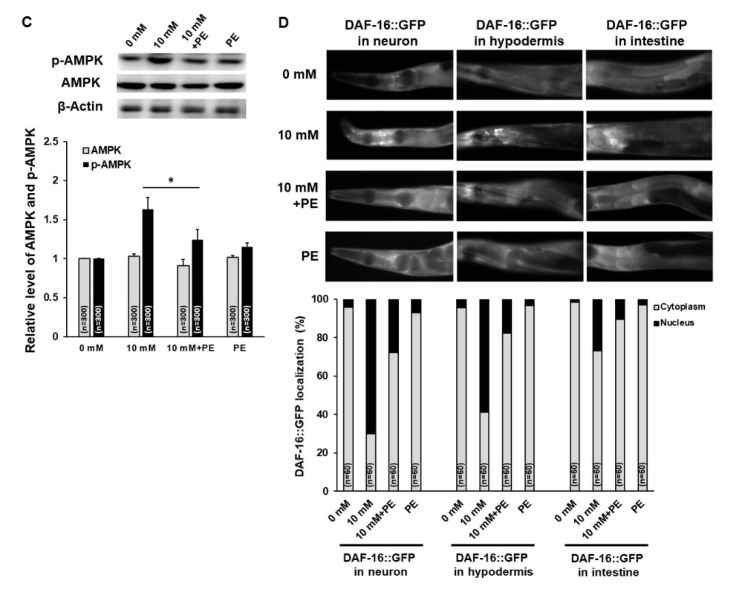
Phosphoethanolamine (PE) supplementation mitigates mitochondrial stress response and activation of AMPK and DAF-16 owing to caffeine intake in *Caenorhabditis elegans*. (**A**,**B**) HSP-6::GFP and GST-4::GFP transgenic animals were synchronized at the L4-stage and fed either 0 or 10 mM of caffeine for 24 h at 20 °C with PE supplementation, and the respective fusion proteins were observed under fluorescence microscopy and quantified using ImageJ analysis. Statistical significance was calculated using one-way ANOVA. *, *p* < 0.05. Error bars represent standard deviation (SD). (**C**) Western blot analysis of phospho-AMPK protein levels in animals fed caffeine with PE supplementation. Respective AMPK and phospho-AMPK band intensities were normalized against those of β-Actin in the same lane. Then, the normalized AMPK and phospho-AMPK band intensities were converted to a relative value in comparison to that of the normalized AMPK and phospho-AMPK band intensities at 0 mM, as shown in the graph; values are mean ± SD of three biological replicates of lysates from 300 animals. Statistical significance was calculated using the Student’s *t*-test. *, *p* < 0.05. (**D**) Images show the DAF-16::GFP expression patterns of animals fed caffeine supplemented with PE in the respective tissues. DAF-16::GFP transgenic animals were synchronized at the L4-stage and were fed either 0 or 10 mM of caffeine for 24 h at 20 °C with PE supplementation, and the fusion protein was observed under fluorescence microscopy. The graph indicates the percentage of animals that show a distribution in the subcellular localization of DAF-16 in the respective tissues.

Next, we determined whether PE supplementation alleviates the increased activation of AMPK and DAF-16 by reducing mitochondrial stress response under caffeine intake conditions. First, we measured the changes in mitochondrial stress response with caffeine intake and PE supplementation through observations of *hsp-6::GFP* and *gst-4::GFP* transgenic animals, and found significant reductions in the expression of both transgenes with PE supplementation (Figure 6A,B). These results indicated that PE supplementation decreased the mitochondrial stress response induced by caffeine intake. We further examined whether PE supplementation could suppress the activity of AMPK and DAF-16 induced by caffeine intake. We found that the protein level of phospho-AMPK was indeed significantly reduced in wild-type animals with PE supplementation (Figure 6C). This result indicated that dietary intake of PE can decrease the level of phospho-AMPK in caffeine-fed animals. Furthermore, we also examined whether the nuclear localization of DAF-16 is suppressed by PE supplementation using transgenic animals expressing a *daf-16::GFP* transgene (Figure 6D). Notably, animals fed caffeine with PE supplementation showed a reduced level of DAF-16 nuclear accumulation in all three tissues, including the neurons, hypodermis, and intestine. These results indicated that the reduction in PE levels by caffeine intake likely modulated DAF-16::GFP nuclear localization. Importantly, these findings suggest that the cellular PE level is one of the critical factors that regulate the signaling pathways triggered by a mitochondrial stress response in organisms.

### 3.6. PE Supplementation Suppresses the Effects of Caffeine Intake on Lipid Metabolism in C. elegans

**Figure 7:**
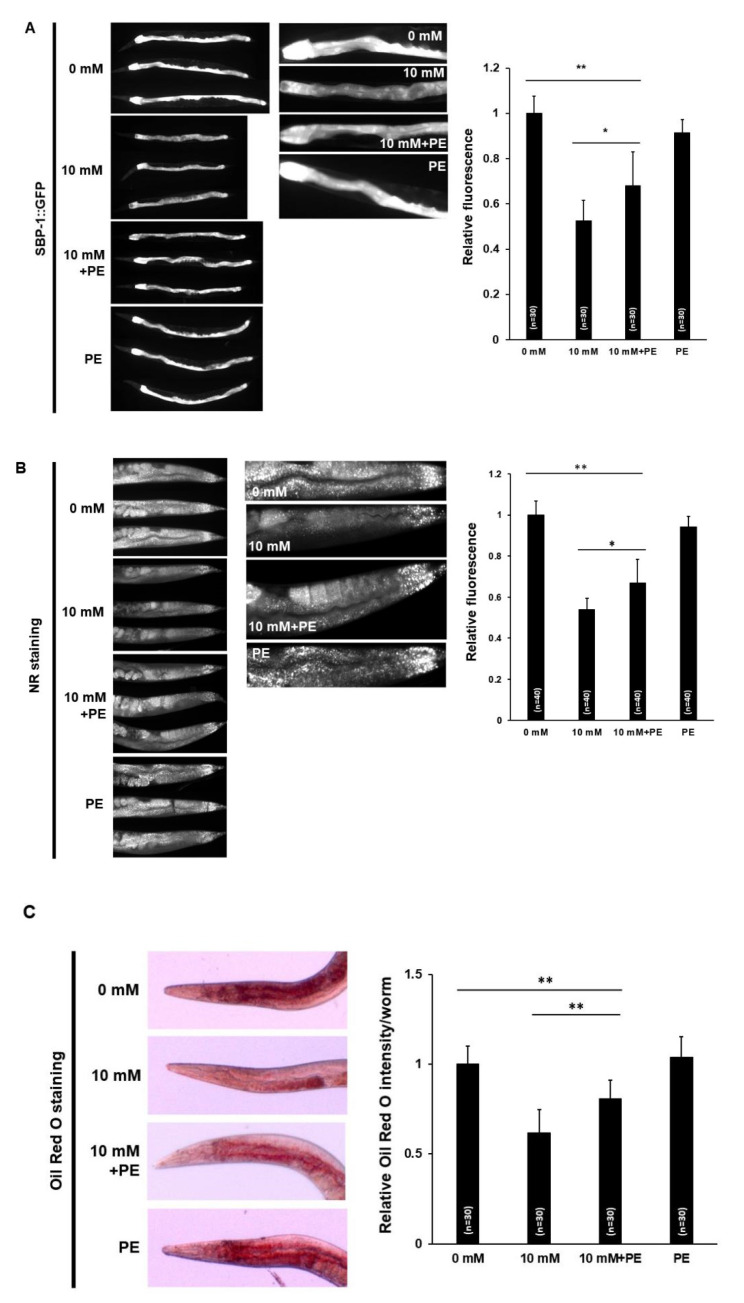
Phosphoethanolamine (PE) supplementation improves lipogenesis and fat storage affected by caffeine intake in *Caenorhabditis elegans*. (**A**) SBP-1::GFP transgenic animals synchronized at the L4-stage were exposed to caffeine with PE supplementation for 24 h at 20 °C. An increased level of SBP-1::GFP in the intestine was observed in animals fed caffeine with PE supplementation. Statistical significance was calculated using one-way ANOVA. *, *p* < 0.05. **, *p* < 0.01. Error bars represent standard deviation (SD). (**B**,**C**) Animals fed caffeine supplemented with PE showed partially increased lipid content as quantified by both Nile Red (NR) and Oil Red O staining. Wild-type animals synchronized at the L4-stage were exposed to caffeine (10 mM) with PE supplementation for 24 h at 20 °C. The graphs show the relative NR fluorescence or Oil Red O intensity of animals fed caffeine supplemented with PE compared to those of caffeine-fed animals. Statistical significance was calculated using one-way ANOVA. *, *p* < 0.05. **, *p* < 0.01. Error bars represent SD.

Next, we attempted to elucidate whether the effects of caffeine intake on lipid metabolism are suppressed by PE supplementation. We first examined the expression level of *sbp-1::GFP* in transgenic animals supplemented with PE. The expression level of *sbp-1::GFP* in animals fed caffeine with PE supplementation was increased significantly; however, the level was moderate (Figure 7A). This result suggested that the low PE level partially contributed to the decreased expression of *sbp-1::GFP* in animals fed caffeine, and other unknown factors are required to fully rescue the decreased lipogenesis owing to caffeine intake.

**Figure 8:**
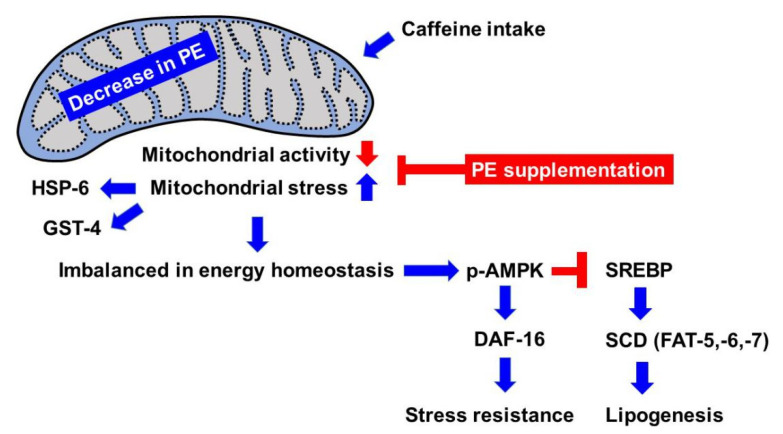
Model of the beneficial effects of phosphoethanolamine (PE) supplementation on mitochondrial stress and lipogenesis owing to caffeine intake in *Caenorhabditis elegans*.

We also examined the effect of PE supplementation on fat storage in animals fed caffeine. A significant improvement in fat storage was observed in animals fed caffeine supplemented with PE, although the level was not similar to that of the animals fed a caffeine-free diet (Figure 7B). This result suggested that the decrease in PE level because of caffeine intake in part reduced fat storage through lipogenesis. Taken together, we propose that caffeine intake causes a reduction in the cellular level of PE, which activates phospho-AMPK and DAF-16 nuclear localization, and suppresses lipogenesis, which is mediated by the increased mitochondrial stress response (Figure 8). In summary, our findings indicate that dietary PE supplementation in caffeine-fed animals bestows beneficial effects on mitochondrial integrity disrupted owing to caffeine intake.

## 4. Discussion

Environment and nutritional status have been explored as important regulators in developmental processes and metabolic pathways [50,51,52]. The effects of caffeine, the most widely consumed diet component, have been studied in a *C. elegans* model to understand its physiological roles both at the molecular and organismal level [10,11,12,13,14,15,16,53,54]. Previous studies have shown age- and dose-dependent adverse and beneficial effects of caffeine intake. Intake of a higher dose of caffeine (>10 mM) at the earlier developmental stage of animals showed adverse effects, such as developmental arrest, activation of stress-response pathways, and stimulation of food-avoidance behavior [10,11,12], whereas animals treated with a low dose of caffeine (<10 mM) at the later developmental stage generally showed beneficial effects. Therefore, we examined the physiological effects of caffeine ingestion with 10 mM caffeine feeding at the fourth larval stage, and observed these effects in adult-stage *C. elegans* in this study. At this stage of *C. elegans*, most developmental processes are completed, and thus, it is convenient to investigate caffeine’s effects on metabolism and overall health. We also recently reported the reduced fertility caused by defective oocytes and eggshell integrity resulting from the decreased production of yolk protein owing to caffeine intake in adult-stage *C. elegans* [13], thereby suggesting that caffeine modulates metabolism. Here, we further examined the effects of caffeine intake on metabolism by analyzing lipid composition in caffeine-fed animals because caffeine intake is known to be closely linked to lipid metabolism [4,5,6,7]. We found that caffeine intake significantly reduced the level of PE, leading to a decrease in mitochondrial activity and disruption in mitochondrial morphology. Notably, upon PE supplementation, these adverse effects of caffeine intake on mitochondrial functions were partially alleviated.

Phospholipids can have different structures in an aqueous environment depending on their shapes [55]. For example, phosphatidylcholine has a cylinder shape that can assemble into lamellar phase. On the other hand, phosphatidylethanolamine has a cone shape that can assemble into hexagonal phase. PE is the second-most abundant metabolite in membrane phospholipids and an intermediate of phosphatidylethanolamine biosynthesis [56,57]. Synthesized phosphatidylethanolamine is abundant in the mitochondrial inner membrane [26,27], and it can form a hexagonal phase that is thought to play a role in membrane fusion events, which are important for mitochondrial function [58,59]. These findings suggest that PE utilization is essential to mitochondrial integrity. Mitochondria are important organelles in the cells for maintaining cellular homeostasis, such as ATP generation via oxidative phosphorylation and regulation of Ca^2+^ levels and multiple metabolic pathways of lipids, amino acids, and iron-sulfur clusters [60,61,62]. In addition, mitochondria have vital activities, such as stress responses, detoxification, and immune responses [63,64]. Given their crucial roles in cell physiology, it is obvious that mitochondria are the first responders to various stressors that challenge homeostasis in the organism. Consistently, the low PE level owing to caffeine intake observed in this study considerably influenced mitochondrial activity and morphology. This finding was supported by the alleviation of the adverse effects of caffeine intake with PE supplementation. Furthermore, we found that low PE levels caused alterations in mitochondrial stress response and lipogenesis, which were also improved upon PE supplementation; although they were not fully recovered, the effect was significantly reduced. Therefore, our findings confirmed the roles of PE supplementation in mitochondrial functions in caffeine-fed animals.

We also found not only a reduction in PE but also an increase in arachidonic acid (AA) with caffeine intake in adult-stage *C. elegans* (Figure 1). This finding suggests the possibility that either reduction in PE or increase in AA or both can cause the effects of caffeine intake observed in this study. In addition, AA is also the structural phospholipid in the cell membrane [65]. In fact, the effects on caffeine-fed animals were partially suppressed by PE supplementation, which may be attributed to the residual increased level of AA. Therefore, the effects of the increased AA level remain to be determined along with those of the low PE level in caffeine-fed animals. Furthermore, it is interesting to note that PDE inhibitors induce the lipolysis pathway in mice [66]. Three main phospholipases, including phospholipase A2, phospholipase C, and phospholipase D, act on phospholipids to produce esterified AA [67]. Considering that caffeine is one of the PDE inhibitors [68], phospholipids can be catalyzed by phospholipases to form AA with caffeine intake [67,69], and thus, increase the AA level. It would be worthwhile to postulate how caffeine intake reduces the level of PE. It has been reported that caffeine can directly interact with unsaturated lipid membranes and locate at the head group–tail group interface of the bilayers [70]. In this sense, it is possible that caffeine interacts with PE and neutralizes the caffeine-induced effects although the direct interaction between caffeine and PE remains to be determined. In this study, on supplementation with ETA, the effects of caffeine intake were also improved as with PE, suggesting that the PE biosynthesis pathway is intact. Therefore, the availability of precursors of ETA in the PE synthesis pathway may be one possible explanation for the low level of PE in caffeine-fed animals. For example, serine is an important precursor of ETA [71]. To elucidate this possibility, amino acid metabolism with caffeine intake needs to be further analyzed.

Previous studies have shown that a low dose of caffeine inhibits oxidative stress, neuroinflammation, and synaptic dysfunction in both a lipopolysaccharide-induced mouse model and a rat model [72,73], suggesting that caffeine appears to be a potent antioxidant and has a protective effect on the oxidative stress response at a low dose. Our results showed that caffeine intake induced the mitochondrial oxidative stress response, showing increased expression of the *hsp-6* gene, and the *gst-4* gene encoding an antioxidant enzyme (Figure 3A,B), suggesting the production of reactive oxygen species (ROS) in caffeine-fed animals. Furthermore, we examined mitochondrial ROS generation using CellROX Green, which showed increased ROS production in mitochondria by caffeine intake (Figures S1 and S2), indicating that caffeine intake increases mitochondrial ROS and oxidative stress responses.

In this study, we revealed the adverse effects of caffeine intake on mitochondrial integrity and lipid metabolism through AMP-activated protein kinase (AMPK) activation in a *C. elegans* model. In contrast, it has been previously suggested that a low dose (1–5 mM) of caffeine intake increases mitochondrial biogenesis in myotubes in primary cultured myotubes [74]. Although there are contradictions in the dose-dependent effect of caffeine intake in different systems, it is certain that the mode of action owing to caffeine intake is associated with mitochondrial modulation. As an energy-sensing signaling pathway, AMPK responds to the decreased cellular energy and is activated by phosphorylation [75]. It was reported that DAF-16, the forkhead box transcription factor class O (FoxO) ortholog of *C. elegans*, is the downstream target of phospho-AMPK in oxidative stress resistance and longevity [36]. Phospho-AMPK also regulates lipogenesis through the suppression of SREBP in mice [41,42], and reduces lipid accumulation in mammalian liver cells [76]. In our study, the expression of *sbp-1*, a master transcriptional regulator of lipid synthesis, was decreased in caffeine-fed animals in which AMPK was activated (Figure 4). These findings are consistent with our results, which show that caffeine intake activates AMPK, and thus DAF-16, but suppresses SREBP, which induces stress resistance and reduces lipogenesis, respectively (Figure 8).

It is proposed that dietary phospholipids, as a mixture or as individual phospholipids, have beneficial effects on human health [77]. Soybean, eggs, and milk are excellent sources of PE [78], and we can consume PE daily through these foods. As shown in this study, PE supplementation can mitigate the adverse effects of caffeine intake. Therefore, under certain circumstances, PE is an important dietary supplement for good health. Finally, it is remarkable that a single component, PE, can improve mitochondrial integrity, and thus, its functions.

## Supplementary Materials

The following are available online at https://www.mdpi.com/2072-6643/12/11/3348/s1, Figure S1: The effect of caffeine intake on mitochondrial-specific superoxide levels, Figure S2: The effect of caffeine intake on mitochondrial ROS generation, Figure S3: The effect of caffeine intake with phosphoethanolamine (PE) supplementation on mitochondrial activity, Table S1: Analysis of lipid composition after caffeine treatment through GC-TOF-MS chromatography in *Caenorhabditis elegans*, Table S2: Statistical analysis by ANOVA.
