# Supplementary material for: Effects of Phosphoethanolamine Supplementation on Mitochondrial Activity and Lipogenesis in a Caffeine Ingestion Caenorhabditis elegans Model
Source: Nutrients. 2020 Oct 30;12(11):3348. doi: 10.3390/nu12113348 (PMC7694071; doi:10.3390/nu12113348)
Supplement: Supplementary file 1 [file nutrients-12-03348-s001.zip › supplementary Table S2.docx]

**Supplementary Table S2. Statistical analysis by ANOVA**

Table 1. Statistical analysis in Fig 5A by one-way ANOVA

| **Groups** | **Count** | **Sum** | **Average** | **Variance** | **p-value** |
| --- | --- | --- | --- | --- | --- |
| 10 mM | 46 | 4.53E+08 | 37779244 | 5.34E+13 |  |
| 10 mM+PE | 42 | 5.72E+08 | 47688052 | 1.13E+14 | 0.01422 |

Table 2. Statistical analysis in Fig 5E by one-way ANOVA

| **Groups** | **Count** | **Sum** | **Average** | **Variance** | **p-value** |
| --- | --- | --- | --- | --- | --- |
| 10 mM | 30 | 8.61 | 0.7175 | 0.010239 |  |
| 10 mM+ETA | 30 | 9.74 | 0.811667 | 0.008488 | 0.026195 |

Table 3. Statistical analysis in Fig 6A by one-way ANOVA

| **Groups** | **Count** | **Sum** | **Average** | **Variance** | **p-value** |
| --- | --- | --- | --- | --- | --- |
| 10 mM | 30 | 29.12324 | 1.456162 | 0.043549 |  |
| 10 mM+PE | 30 | 21.05416 | 1.052708 | 0.022718 | 2.39E-08 |

Table 4. Statistical analysis in Fig 6B by one-way ANOVA

| **Groups** | **Count** | **Sum** | **Average** | **Variance** | **p-value** |
| --- | --- | --- | --- | --- | --- |
| 10 mM | 40 | 43438470 | 2714904 | 1.62E+11 |  |
| 10 mM+PE | 50 | 36514961 | 1921840 | 1.84E+11 | 3.09E-06 |

Table 5. Statistical analysis in Fig 7A by one-way ANOVA

| **Groups** | **Count** | **Sum** | **Average** | **Variance** | **p-value** |
| --- | --- | --- | --- | --- | --- |
| 10 mM | 30 | 5.78712 | 0.526102 | 0.002235 |  |
| 10 mM+PE | 30 | 8.160024 | 0.680002 | 0.005664 | 9.17E-06 |

| **Groups** | **Count** | **Sum** | **Average** | **Variance** | **p-value** |
| --- | --- | --- | --- | --- | --- |
| 0 mM | 30 | 10.00688 | 0.855938 | 0.004392 |  |
| 10 mM+PE | 30 | 8.160024 | 0.680002 | 0.005664 | 5.3E-04 |

Table 6. Statistical analysis in Fig 7B by one-way ANOVA

| **Groups** | **Count** | **Sum** | **Average** | **Variance** | **p-value** |
| --- | --- | --- | --- | --- | --- |
| 10 mM | 40 | 11.01324 | 0.555938 | 0.005231 |  |
| 10 mM+PE | 40 | 14.79538 | 0.752199 | 0.004021 | 3.6E-05 |

| **Groups** | **Count** | **Sum** | **Average** | **Variance** | **p-value** |
| --- | --- | --- | --- | --- | --- |
| 0 mM | 40 | 20.00688 | 1.455938 | 0.003865 |  |
| 10 mM+PE | 40 | 14.79538 | 0.752199 | 0.004021 | 4.6E-05 |

Table 7. Statistical analysis in Fig 7C by one-way ANOVA

| **Groups** | **Count** | **Sum** | **Average** | **Variance** | **p-value** |
| --- | --- | --- | --- | --- | --- |
| 10 mM | 30 | 10.75006 | 0.554657 | 0.004392 |  |
| 10 mM+PE | 30 | 13.56482 | 0.655299 | 0.003215 | 3.1E-03 |

| **Groups** | **Count** | **Sum** | **Average** | **Variance** | **p-value** |
| --- | --- | --- | --- | --- | --- |
| 0 mM | 30 | 21.02547 | 1.533762 | 0.002657 |  |
| 10 mM+PE | 30 | 13.56482 | 0.655299 | 0.003215 | 5.7E-03 |
