# Supplementary figures and images for: Effects of Phosphoethanolamine Supplementation on Mitochondrial Activity and Lipogenesis in a Caffeine Ingestion Caenorhabditis elegans Model
Source: Nutrients. 2020 Oct 30;12(11):3348. doi: 10.3390/nu12113348 (PMC7694071; doi:10.3390/nu12113348)

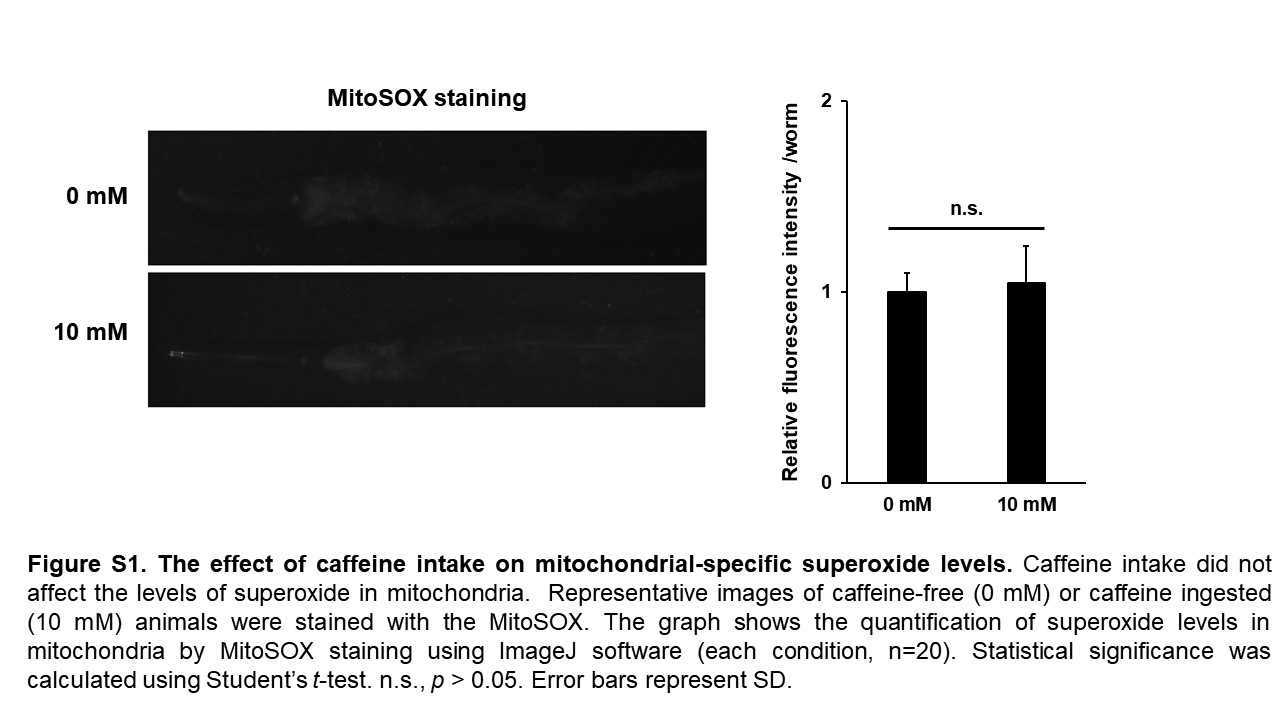

Supplement: Supplementary file 1 [file nutrients-12-03348-s001.zip › S1.jpg]

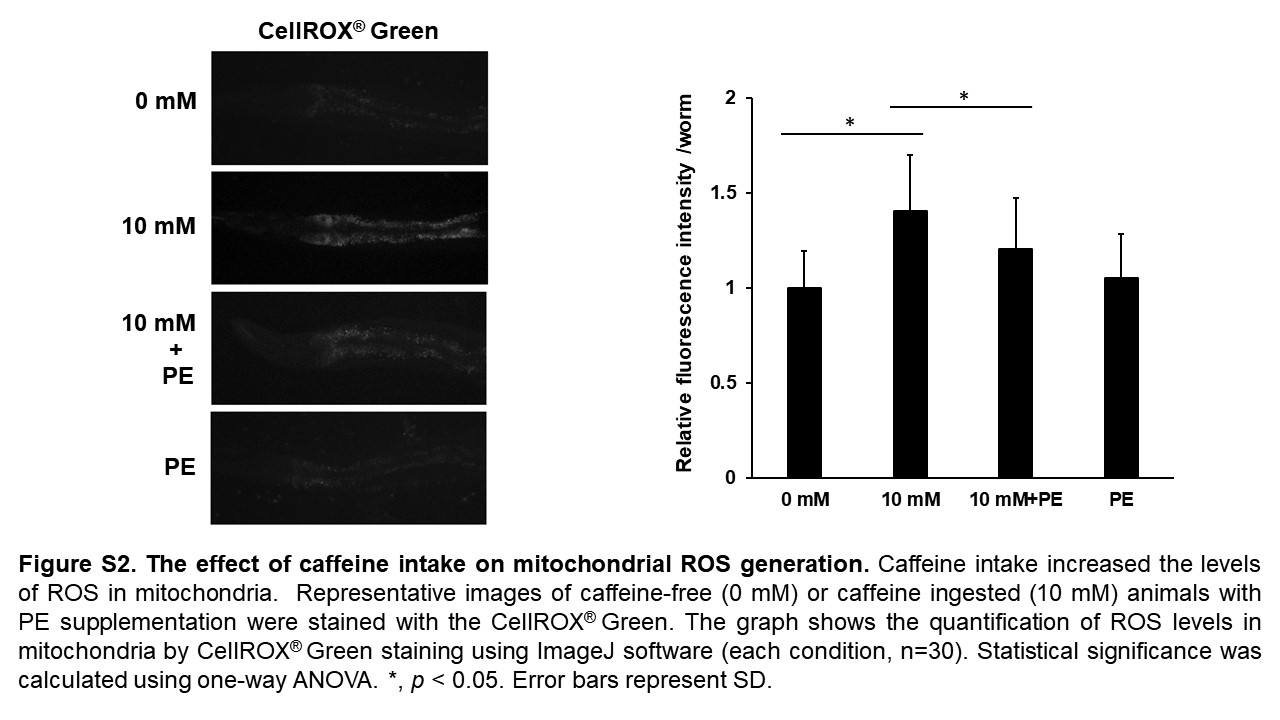

Supplement: Supplementary file 1 [file nutrients-12-03348-s001.zip › S2.jpg]

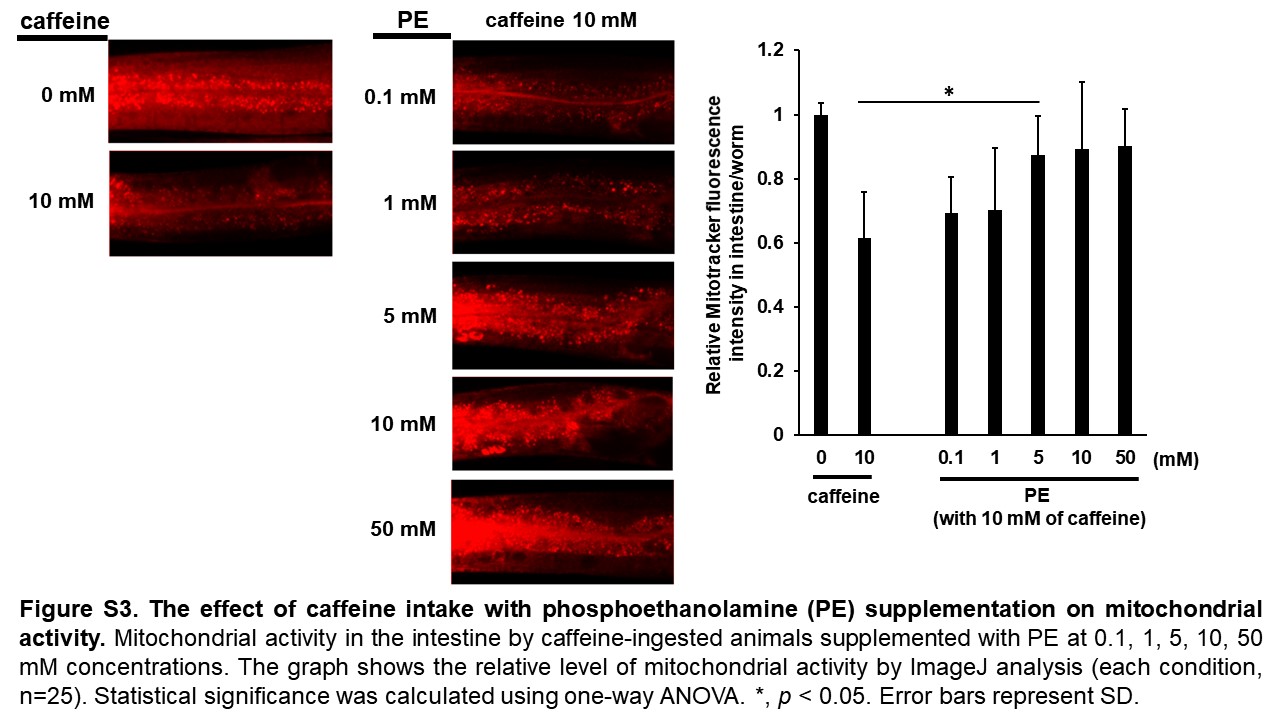

Supplement: Supplementary file 1 [file nutrients-12-03348-s001.zip › S3.jpg]
